# Carrageenan nasal spray may double the rate of recovery from coronavirus and influenza virus infections: Re‐analysis of randomized trial data

**DOI:** 10.1002/prp2.810

**Published:** 2021-06-14

**Authors:** Harri Hemilä, Elizabeth Chalker

**Affiliations:** ^1^ Department of Public Health University of Helsinki Helsinki Finland; ^2^ School of Public Health University of Sydney Sydney Australia

**Keywords:** common cold, iota‐carrageenan, meta‐analysis, quantile treatment effect, randomized trial, rhinovirus, SARS‐CoV‐2

## Abstract

In this individual patient data meta‐analysis we examined datasets of two randomized placebo‐controlled trials which investigated the effect of nasal carrageenan separately on children and adults. In both trials, iota‐carrageenan was administered nasally three times per day for 7 days for patients with the common cold and follow‐up lasted for 21 days. We used Cox regression to estimate the effect of carrageenan on recovery rate. We also used quantile regression to calculate the effect of carrageenan on colds of differing lengths. Nasal carrageenan increased the recovery rate from all colds by 54% (95% CI 15%–105%; *p* = .003). The increase in recovery rate was 139% for coronavirus infections, 119% for influenza A infections, and 70% for rhinovirus infections. The mean duration of all colds in the placebo groups of the first four quintiles were 4.0, 6.8, 8.8, and 13.7 days, respectively. The fifth quintile contained patients with censored data. The 13.7‐day colds were shortened by 3.8 days (28% reduction), and 8.8‐day colds by 1.3 days (15% reduction). Carrageenan had no meaningful effect on shorter colds. In the placebo group, 21 patients had colds lasting over 20 days, compared with six patients in the carrageenan group, which corresponds to a 71% (*p* = .003) reduction in the risk of longer colds. Given that carrageenan has an effect on diverse virus groups, and effects at the clinical level on two old coronaviruses, it seems plausible that carrageenan may have an effect on COVID‐19. Further research on nasal iota‐carrageenan is warranted.

## Graphical abstract

Nasal carrageenan may help against coronavirus, influenza and rhinovirus infections.

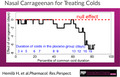

**Abbreviations**
CI: confidence interval
IPD: individual‐patient data
NNT: number needed to treat
RCT: randomized trials
RR: risk ratio

**What is already known about this subject**

‐ Mucosally administered carrageenan has decreased mortality of mice infected by influenza A and by herpes simplex.
‐ Laboratory studies have indicated that carrageenan can protect against SARS‐CoV‐2 in cell cultures.
‐ Four RCTs have indicated that nasal iota‐carrageenan may alleviate common cold symptoms.

**What this study adds**

‐ Nasal carrageenan increased the recovery rate of coronavirus, influenza A, and rhinovirus infections.
‐ Quantile treatment effect appears a useful measure of treatment effects on the duration of infections.

## 1 INTRODUCTION

Carrageenan is a sulfated polysaccharide extracted from red seaweed, commonly known as Irish Moss.^1^, ^2^, ^3^ Since the 1980s, carrageenan has been shown to prevent infections due to several viruses in cell cultures, including influenza viruses, coronavirus OC43, rhinoviruses, and coxsackievirus.^4^, ^5^, ^6^, ^7^, ^8^, ^9^, ^10^, ^11^ A few very recent studies showed that carrageenan can inhibit the replication of the SARS‐CoV‐2 virus.^12^, ^13^, ^14^, ^15^ Mortality of mice infected by influenza A^15^, ^16^, ^17^ and by herpes simplex^18^ was significantly decreased by mucosally administered carrageenan, which indicates that the effects on respiratory viruses are not limited to cell cultures.

Carrageenan has been used as a food component for decades and is classified by the FDA as “generally regarded as safe” [GRAS].^19^ Many concerns about potential harms of carrageenan have been shown to be unfounded and are explained, for example, by confusing carrageenan with polygeenan and using inappropriate biological model systems.^19^, ^20^, ^21^ A recent review concluded that animal studies have found dietary carrageenan to be safe in that it is not a carcinogen or tumor promoter, nor does it have developmental, reproductive, or genotoxic effects.^19^ The European Food Safety Authority states that “no adverse effects have been detected in chronic toxicity studies with carrageenan in rats up to 7500 mg/kg bw per day”.^22^ For a 70 kg person, this corresponds to 525 g per day. A recent study with mice and rabbits indicated that nasal and pulmonary administration of iota‐carrageenan did not cause acute adverse effects.^23^

Three randomized trials (RCT) with adults^24^, ^25^, ^26^ and one trial with children^27^ found that nasally administered iota‐carrageenan shortened and alleviated common cold symptoms. The carrageenan dose in these trials was 0.001 g/day administered nasally for about one week and so potential concerns about very high oral doses for decades are not pertinent. An individual‐patient data (IPD) meta‐analysis pooled the results of one adult trial^25^ and the child trial,^27^ for which cold duration data were available, and concluded that there was evidence that iota‐carrageenan shortened colds caused by coronaviruses OC43 and 229E, influenza A virus, and rhinoviruses.^28^ The mean duration of all virus‐positive colds was reported to be 1.9 days shorter in the iota‐carrageenan groups.^28^ However, several patients did not recover by the end of the follow‐up and therefore the mean duration is not an appropriate measure of effect. Furthermore, when assessing the effects of interventions on common cold duration, relative effect estimates such as percentages are preferable to days shortened.^29^ In this reanalysis of the IPD of the two trials^25^, ^27^ we estimate the effect of carrageenan on the common cold using survival analysis and quantile regression, both of which are not limited by some patients not recovering during the follow‐up period.

## 2 MATERIALS AND METHODS

The Ludvig trial with adults^25^ and the Fazekas trial with children^27^ are described in detail in the trial reports. In brief, both trials were randomized, double‐blind, placebo‐controlled trials, carried out in Vienna, Austria. For enrollment, participants were required to have mild to moderate common cold symptoms and the duration of colds was not allowed to be longer than 48 h for adults,^25^ and 36 h for children.^28^ In both trials, the randomization list was prepared by a third party and patients were randomly assigned using a permuted block schedule (size four).^28^ The carrageenan spray and the placebo spray were indistinguishable. A single dose of 0.14 ml of nasal spray (0.12% iota‐carrageenan) was administered to both nostrils three times per day for 7 days in both trials. Thus, the total daily dose of iota‐carrageenan was 1.0 mg.

The biological effect of carrageenan appears to prevent the virus from binding to cell surfaces or penetrating the cells,^1^, ^2^, ^3^ so the pooled IPD analysis^28^ was limited to virus‐positive participants. In the Ludvig trial, 59 of 102 participants in the carrageenan group and 59 of 101 participants in the placebo group were virus positive.^25^ In the Fazekas trial, 67 of 76 participants in the carrageenan group and 69 of 77 participants in the placebo group were virus positive.^27^ Thus, the proportion of participants who were virus positive was very similar in the treatment arms within both trials.

Survival curves for the combined IPD of all virus‐positive participants in the two trials^25^, ^27^ were published as figure 2 in the previous meta‐analysis.^28^ Survival curves for the colds caused by coronaviruses OC43 and 229E, influenza A virus, and rhinoviruses were published in figure 5 of Ref. [28]. Figures 2 and 5 of Ref. [28] reported intention‐to‐treat [ITT] and per‐protocol data. Since the ITT analysis is generally preferable,^30^, ^31^ we used the ITT data for infected participants in our estimation of the carrageenan effect.

To regenerate the dataset, we downloaded the two figures and measured the height of the steps in the ITT curves using a graphics program and transformed the step heights to the number of patients who recovered on each day during the follow‐up (see details in the Supplement). There were problems in regenerating the data from the curves for rhinovirus colds, but these were resolved after receiving data from Dr. Eva Prieschl‐Grassauer. The rate of recurrence of colds was calculated from figure 3 of Ref. [28] for all colds, and from figure 6 of Ref. [28] for the virus‐specific data.

In this study, we analyze two outcomes^28^: (1) duration of the common cold defined as the time until the last day with common cold symptoms and (2) recurrence of common cold symptoms after the patient reported having been without symptoms for at least 1 day.

We used two approaches to estimate the effect of iota‐carrageenan on common cold duration: survival analysis and quantile regression. We used the *coxph* procedure of the *survival* package of the R‐project to calculate the risk ratio (RR) for the recovery from the common cold and its 95% confidence interval (CI).^32^, ^33^ For ties, we used the *exact* option, except for time‐dependent survival analysis, for which we used the *Efron* option. We used the likelihood ratio test to calculate the P‐value for the effect of carrageenan.

We also used quantile regression to analyze quantile treatment effects.^34^, ^35^, ^36^ A similar approach was recently used to analyze the therapeutic effect of vitamin C on SARS‐Cov‐2 infection.^37^ Since there were censored data, we used the *crq* procedure of the *quantreg* package of R to calculate the 95% CI for the quantile treatment effect for 80th percentile level using the *PengHuang* option.^35^, ^36^ To estimate the effect of carrageenan on the mean duration of colds within quintiles of the duration distribution, we calculated the mean durations for the 1st to 4th quintiles, but not for the 5th quintile because the last included the censored observations. To estimate the effect of carrageenan on the mean duration within the quintiles, we calculated the days shortened as the difference between the mean durations, and we also calculated the corresponding effect in percentages, which has been shown to be a superior measure compared with the absolute difference in days.^29^

To calculate the risk ratio (RR) for the occurrence of long colds (>20 days) and for the recurrence of cold symptoms after the patient had been without symptoms, and their 95% CI, we used the *riskratio* procedure of the *fmsb* package.^38^ We present two‐tailed *p*‐values. See supplementary file for printouts of the statistical calculations.

## 3 RESULTS

In our analysis, we used the pooled IPD from two iota‐carrageenan randomized trials.^25^, ^27^ The mean ages of participants in the trials were 5 years^27^ and 33 years^25^ and the sex of participants was relatively balanced in both trials. In the iota‐carrageenan groups of the two trials, there were 126 common cold patients with virus‐positive colds, and in the placebo groups there were 128 participants.

**FIGURE 1:**
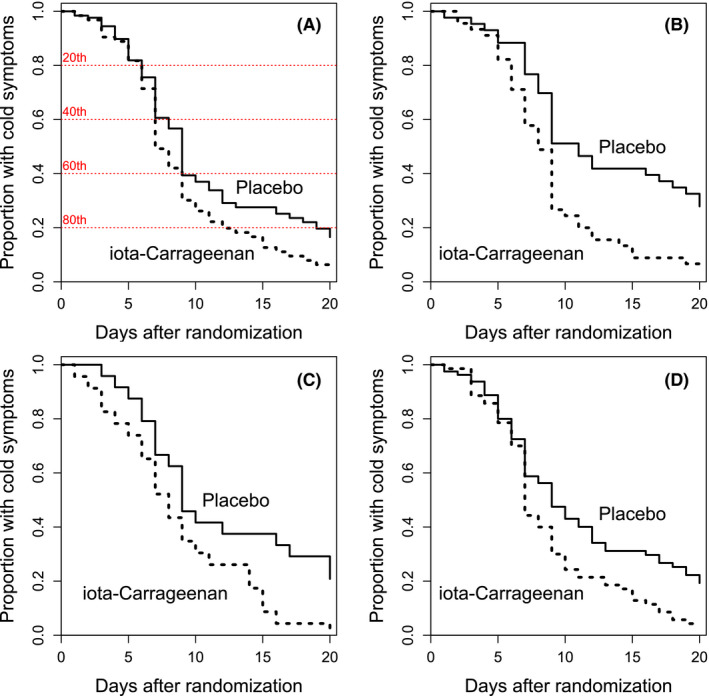
The effect of nasal iota‐carrageenan on the recovery from common cold episodes caused by any virus (A), coronavirus OC43 or 229E (B), influenza A (C), and rhinovirus (D). The ITT survival curves published in figures 2 and 5 of Ref. [28] were measured and datasets were regenerated for the current analysis; see the Supplementary file. In the curves of the figure, the size of the steps downwards indicates the number of patients who recovered on the particular day. The red horizontal dotted lines indicate the 20th, 40th, 60th, and 80th percentiles of the distribution of common cold duration, starting with the shortest colds from the top downwards; compare with Figure 2

Participants who received nasal carrageenan had a recovery rate greater than those who received placebo with RR = 1.54 (Figure 1A). Separate recovery data were available for coronaviruses OC43 or 229E, influenza A virus, and rhinoviruses. The reproduced survival curves for these three virus groups are shown in Figure 1B–D. Carrageenan increased the recovery rate from coronavirus infections by RR = 2.39, from influenza A infections by RR = 2.19, and from rhinovirus infections by RR = 1.70 (Table 1). The confidence intervals for the three virus groups are widely overlapping, indicating that the findings for all the three virus groups are consistent given the inaccuracy of the findings.

**TABLE 1:** Effect of iota‐carrageenan on the rate of recovery from the common cold

Viruses	Number of patients		Effect of iota‐carrageenan	
	Carrageenan	Placebo	RR (95% CI)	*p*
All viruses^a^	126	128	1.54 (1.15–2.1)	.003
Coronavirus OC43 or 229E	45	43	2.39 (1.43–4.0)	.001
Influenza A	23	24	2.19 (1.12–4.3)	.021
Rhinovirus	70	80	1.70 (1.17–2.5)	.006

RR, risk ratio of recovery comparing iota‐carrageenan versus placebo groups.

^a^ In addition to the three virus groups listed, the “all viruses” group includes patients with influenza B, parainfluenza, respiratory syncytial virus, and metapneumovirus. A few patients had two or more viruses.

For all the virus‐positive colds combined, the pattern of the survival curves indicates different effects of carrageenan on short and long colds (Figure 1A). Over the first 5 days, the recovery rate was very similar in the carrageenan and placebo groups with RR = 0.98 (95% CI 0.55–1.73), but during the follow‐up from day 6 onwards, carrageenan increased the recovery rate by RR = 1.64 (95% CI 1.22–2.2; *p* = .001).

**FIGURE 2:**
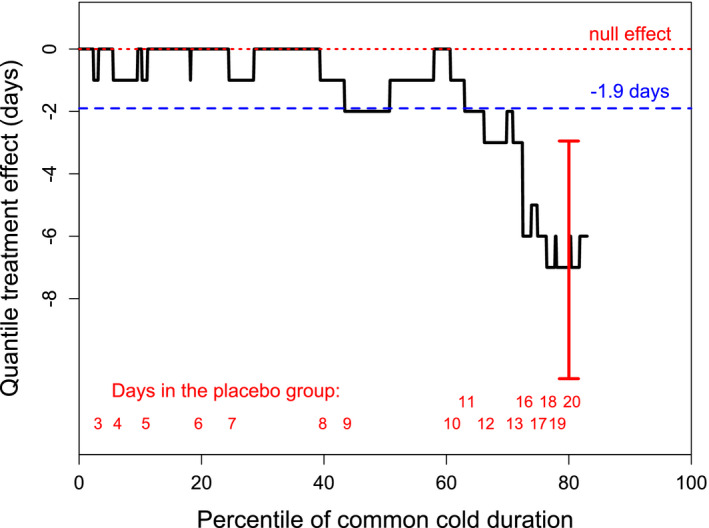
The quantile treatment effect of nasal iota‐carrageenan on the duration of virus‐positive colds as days shortened. The horizontal axis shows the distribution of the duration of colds by percentiles for up to the 83th percentile, after which data in the placebo group was censored, that is, patients did not recover by the end of the follow‐up. The red dotted line indicates the null effect level, and the blue dashed line shows the previously calculated 1.9‐day estimate of effect for carrageenan.^28^ The red figures at the bottom indicate the lowest percentile level for the indicated common cold duration in the placebo group. For example, 9‐day colds cover the percentile range from 43.0 to 60.9 percentiles, which corresponds to 21 patients as the total number of placebo group patients was 128. The red vertical bar indicates the 95% CI of the treatment effect for the 80th percentile

The treatment effect of carrageenan was also analyzed with quantile regression, in which the distribution of common cold duration is set on the horizontal axis as percentiles (Figure 2). The continuous black line shows the carrageenan treatment effect by the percentile levels. The effect of carrageenan is seen to be heterogeneous. The red dotted line indicates the null effect level and the blue dashed line shows the previously calculated effect estimate of 1.9 days,^28^ which exaggerates the effect of carrageenan for short colds, but underestimates the effect for long colds. All percentiles from 0 to 43 had an effect estimate below 1.9 days, whereas all percentiles from 71 to 83 had an effect estimate above 1.9 days. Thus, over half the observations appear inconsistent with the calculated 1.9‐day effect.^28^ In the 80th percentile of patients in the placebo group, colds lasted for 19 days and iota‐carrageenan shortened them by 7 days (95% CI 10.6–2.9 days; *p* = .001). This corresponds to a 37% shortening of colds at the 80th percentile level. The vertical bar shows the 95% CI for the 80th percentile point, which is far from the 1.9‐day estimate (Figure 2).

**FIGURE 3:**
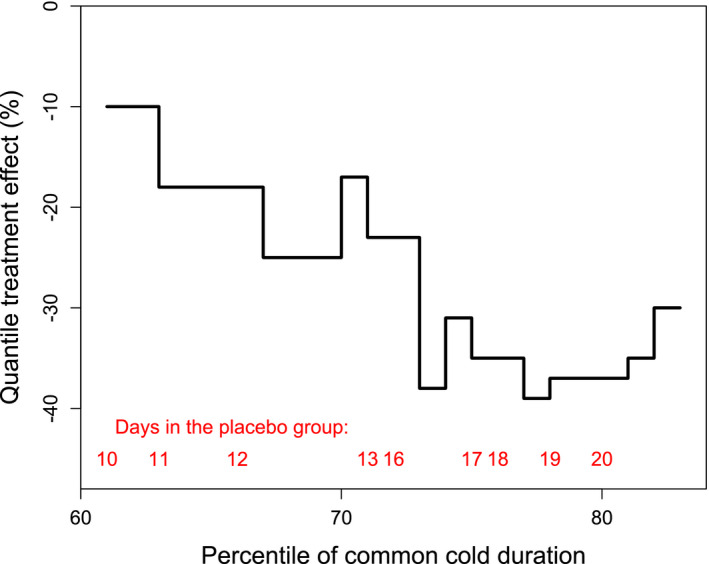
The quantile treatment effect of nasal iota‐carrageenan on the duration of virus‐positive colds as a relative effect in percentages. The horizontal axis shows the distribution of the duration of colds by percentiles from the 60th up to the 83th percentile, after which data in the placebo group were censored, that is, 21 patients in the placebo group did not recover by the end of the follow‐up

Figure 3 shows the quantile treatment effect on the relative scale, which adjusts for the variation in the duration in the placebo group.^29^ Compared with colds that lasted 2 to 3 weeks in the placebo group, colds in the carrageenan group were 20% to 40% shorter.

The effects of carrageenan within the quintile ranges of the distribution of common cold duration are shown in Table 2. The effect is calculated both as an absolute effect in days and as a relative effect in percentages. In the 4th quintile, the untreated mean common cold duration of 13.7 days is shortened on average by 3.8 days which corresponds to a 28% reduction in duration. In the 3rd quintile, the untreated common cold duration of 8.8 days is shortened by 1.3 days which corresponds to a 15% reduction. For the two lowest quintiles, there is no meaningful effect from carrageenan treatment.

**TABLE 2:** Effect of iota‐carrageenan on common cold duration by quintiles of the cold distribution

Quintile	Mean duration of colds		Effect of carrageenan	
	Carrageenan	Placebo	Days saved	Percentage shortened
1.	3.80	4.04	0.24	5.9%
2.	6.56	6.81	0.25	3.6%
3.	7.52	8.84	1.32	14.9%
4.	9.84	13.69	3.85	28.1%

The 5th quintile range is not shown since it contains the participants with censored data. The relative scale (percentage shortened) has been shown to be more informative in the analysis of effects on duration.^29^

The effect of carrageenan on the duration of colds was also analyzed as the risk of a person having a cold that lasted for over 20 days, that is the unrecovered censored observations at the end of the follow‐up. In the carrageenan group the risk of a cold lasting for over 20 days was 71% less than in the placebo group (Table 3). Among the patients administered placebo, 16.4% did not recover by the end of the 20‐day follow‐up, whereas just 4.7% of patients administered carrageenan did not recover. This corresponds to a number needed to treat (NNT) of 8.6 (95% CI 5.2–24).

**TABLE 3:** Effect of iota‐carrageenan on the risk of the common cold lasting over 20 days

	Intervention		Effect of carrageenan	
	Carrageenan	Placebo	RR (95% CI)	*p*
Cold duration >20 days	6	21	0.29 (0.12–0.70)	.003
Total in group	126	128		

RR, risk ratio of common cold lasting over 20 days comparing the iota‐carrageenan versus placebo groups.

The effect of iota‐carrageenan on the recurrence of common cold symptoms is shown in Table 4. In all virus‐positive patients, carrageenan reduced the recurrence of cold symptoms by 56%. The point estimates for the effect of carrageenan in the participants with coronavirus, influenza A virus, and rhinovirus are consistent with the overall effect estimate. In all virus‐positive patients, 28.9% of placebo participants had a recurrence of cold symptoms after first recovering, compared with only 12.7% of carrageenan patients. This corresponds to a NNT of 6.2 (95% CI 3.8–16).

**TABLE 4:** Effect of iota‐carrageenan on the risk of common cold symptoms recurring

Viruses	Recurring symptoms/number of patients		Effect of iota‐carrageenan	
	Carrageenan	Placebo	RR (95% CI)	*p*
All viruses^a^	16/126	37/128	0.44 (0.25–0.75)	.002
Coronavirus OC43 or 229E	8/45	19/43	0.40 (0.20–0.82)	.008
Influenza A	3/23	9/24	0.35 (0.10–1.13)	.057
Rhinovirus	6/70	16/80	0.43 (0.17–1.04)	.049

RR, risk ratio of recurrence of common cold symptoms comparing iota‐carrageenan versus placebo groups.

^a^ In addition to the three virus groups listed, the “all viruses” group includes patients with influenza B, parainfluenza, respiratory syncytial virus, and metapneumovirus. A few patients had two or more viruses.

## 4 DISCUSSION

Adults have on average two colds per year and young children six per year. Therefore, potential interventions to shorten and alleviate common cold symptoms are of great public health importance. Previously, zinc lozenges have been shown to shorten the common cold but the composition of the lozenges is crucial for effectiveness.^29^, ^39^, ^40^, ^41^, ^42^ There is also evidence that regular intake of high‐doses of vitamin C shortens colds that occur during the supplementation period,^43^, ^44^, ^45^ and a recent randomized trial found that therapeutic vitamin C increased the recovery rate for outpatient cases of SARS‐CoV‐2 infection.^37^ It seems that the effects of zinc lozenges and vitamin C for the common cold have been ignored not on the basis of evidence from randomized trials, but because of prejudices.^44^, ^45^, ^46^, ^47^

Carrageenan is a more recent potential treatment for the common cold. Laboratory studies indicate that it has an effect on various respiratory virus infections, including those caused by influenza viruses, coronavirus OC43, rhinoviruses, coxsackievirus, and SARS‐CoV‐2,^8^, ^9^, ^10^, ^11^, ^12^, ^13^, ^14^, ^15^, ^16^, ^17^ but possibly not adenovirus.^4^, ^5^ Although laboratory evidence indicating that carrageenan can prevent virus infections traces back to the 1980s, clinical trials on the common cold have been carried out only since 2010.^24^, ^25^, ^26^, ^27^

A previous IPD meta‐analysis of two carrageenan trials^25^, ^27^ calculated that colds were on average 1.9 days shorter in patients administered nasal carrageenan.^28^ However, the meta‐analysis did not take account of the fact that there were censored data for 27 patients which means that they did not recover by the end of the follow‐up (Figure 1A). Therefore, the mean durations are undefined and the calculation of the difference in mean durations is inappropriate. In addition, the meta‐analysis^28^ did not consider the possibility that the effect of carrageenan might be heterogeneous.

In our IPD meta‐analysis of the same two trials^25^, ^27^ we used Cox regression and quantile regression, both of which take into account the censored observations. We found that there is strong evidence of a treatment effect for nasal iota‐carrageenan when colds last over a week or so (Figures 1A and 2; Table 2). Our analysis did not demonstrate an effect on shorter colds. The heterogeneity in the treatment effect indicates that the previously estimated 1.9‐day reduction in common cold duration^28^ poorly captures the effects of nasal carrageenan (Figure 2).

In our analysis, we calculated that nasal carrageenan increased the recovery rate by 54% for all virus‐positive participants during follow‐up (Table 1). This effect is not as large as the approximately 200% increase in recovery rate in five trials with zinc lozenges,^40^ but important nonetheless.

In quantile regression, we found that carrageenan shortened long colds by 15%–28% (Table 2). These can be compared with the 33% average decrease in common cold duration in seven trials with zinc lozenges,^41^ and the roughly 20% decrease in cold duration with high vitamin C doses.^37^, ^44^, ^45^, ^48^ The benefit of nasal carrageenan was seen only on colds lasting over a week or so. However, a 28% shortening of 2‐week colds is a much more important finding than a similar effect for short colds.

The effect of carrageenan on long colds was also analyzed as the risk of the cold lasting for over 20 days. Nasal carrageenan reduced the risk of such long colds by 71% (Table 3). On the basis of this outcome, one in every nine patients benefited from carrageenan.

The apparent benefit against long colds is relevant when considering two further trials on nasal iota‐carrageenan. The first trial by Eccles administered carrageenan just for 4 days.^24^ The second trial by Eccles also administered carrageenan for 4 days, yet patients were allowed to use it for longer; however, there are no data about how long the patients actually used carrageenan in that trial.^26^ Furthermore, the two trials followed the patients for just 7 and 10 days, respectively, while the current analysis over 21 days indicates that the greatest benefits may appear only after 7 days (Figures 1A and 2). Nevertheless, even though ideally the intervention and follow‐up periods should have been longer, the two short‐term trials also found that carrageenan was beneficial. In the first Eccles trial, the total symptom score over days 2–4 was decreased by 26% (*p* = .046),^24^ and in the second, the total symptom score over days 1–4 was decreased by 9% (*p* = .042).^26^ Reduction in the respiratory virus load has also been observed in the carrageenan participants.^24^, ^25^, ^26^, ^27^, ^28^

We also found that carrageenan halved the recurrence of colds during the follow‐up period (Table 4). On the basis of this finding, one in every six patients benefited from carrageenan. While it is not evident whether the recurrence of symptoms was caused by the same virus or by a new virus, halving the occurrence of new cold‐type symptoms in such a large proportion of participants is a clinically relevant finding. Most recurrences occurred after cessation of treatment^28^ and therefore administration for longer than 7 days should be tested in further trials to ascertain whether recurrence may be further reduced.

The common cold is not a homogeneous entity. The majority of common cold symptoms are caused by several different virus types, but the distribution of viruses varies over time and location. In addition, some of the cold‐type symptoms are caused by non‐viral causes such as allergy and mechanical irritation. Nevertheless, as regards SARS‐CoV‐2, the pattern of findings from carrageenan is particularly interesting. Our analysis gives strong direct evidence that carrageenan is effective against two old coronaviruses OC43 or 229E by increasing the recovery rate by 139% and by decreasing the recurrence of cold symptoms by 60%. Even the old coronaviruses have caused severe acute respiratory infections.^49^ Furthermore, the efficacy against rhinovirus and influenza A virus indicates that the effects are nonspecific. This does not necessarily mean that carrageenan is effective against SARS‐CoV‐2; however, the non‐specificity of carrageenan makes it highly plausible that carrageenan will also have an effect on COVID‐19. A very recent controlled trial found that iota‐carrageenan prevented SARS‐CoV‐2 infection in health care workers who were exposed to COVID‐19 patients,^50^ Finally, given the particular concern around long cases of COVID‐19,^51^, ^52^ the proportionally greater effect of carrageenan on long infections seems particularly important.

The primary outcome in our analysis was self‐reported recovery from the common cold in the two included trials.^25^, ^27^ Although some researchers may consider that a subjective outcome such as this one is suboptimal, it is the patient who decides whether to visit a physician to ask for a certificate for sick leave or to take time off work because of illness. Diagnosis of the common cold by virology is not feasible because of the large number of viruses and the variability in the disease states caused by the viruses. Given that patients are familiar with the common cold it seems a more reasonable approach to use self‐diagnosis for clinical research.^53^ Furthermore, the FDA encourages patient‐reported outcomes, because they are not biased by the interpretations of physicians or others.^54^

All four trials on nasal iota‐carrageenan used 3–4 daily doses.^24^, ^25^, ^26^, ^27^ In further research, the dose–response should be investigated by testing a higher frequency of use to identify the level that leads to maximal effects. Similarly, the dose–response for the amount of carrageenan within the single nasal dose should be examined. Since most recurrences of cold symptoms occurred after cessation of treatment, longer administration should also be tested. It is also possible that the length of time between the onset of symptoms and the start of treatment has an impact on the effectiveness. The two trials included in our meta‐analysis specified that treatment was to be started within 36–48 h of symptom onset,^25^, ^28^ whereas the corresponding time limit in zinc lozenge trials has often been 24 h.^42^ Evidently, the effect of the time between the onset of symptoms and the start of carrageenan treatment on the size of the benefit should be investigated in further trials.

Laboratory studies have found that the combination of carrageenan with oseltamivir and zanamivir has synergistic effects against murine influenza.^16^, ^17^ Similarly, it would seem reasonable to examine in a factorial setting the effects of combining carrageenan with zinc lozenges and/or vitamin C since they all seem to have different modes of effect.

In conclusion, we estimate that nasal iota‐carrageenan may increase the recovery rate from the common cold by about 50% and shorten the duration of long colds by about 30%. We did not find a beneficial effect on short colds. If able to be replicated, the findings of this study are important for future treatment options for coronavirus and influenza virus infections. Further research should be carried out to examine the effects of nasal iota‐carrageenan on respiratory virus infections in more detail.

## CONFLICT OF INTEREST

The authors declare that the research was conducted in the absence of any commercial or financial relationships that could be construed as a potential conflict of interest.

## AUTHOR CONTRIBUTIONS

HH planned the study, measured the published survival curves,^28^ entered the data into a spreadsheet and carried out the statistical analysis, and wrote the draft manuscript. EC checked that the entered data were consistent with the published survival curves and participated in the critical revision of the manuscript. Both authors read and approved the final manuscript.

## Supporting information

Supplementary Material

## Data Availability

Data analyzed in this study are available in the Supplement.
