# Supplementary material for: Carrageenan nasal spray may double the rate of recovery from coronavirus and influenza virus infections: Re‐analysis of randomized trial data
Source: Pharmacol Res Perspect. 2021 Jun 14;9(4):e00810. doi: 10.1002/prp2.810 (PMC8204093; doi:10.1002/prp2.810)
Supplement: Supplementary file 1 — Supplementary Material [file PRP2-9-e00810-s001.pdf]

Carrageenan nasal spray may double the rate of recovery from coronavirus and influenza virus infections: re-analysis of randomized trial data

Harri Hemilä[1] and Elizabeth Chalker[2]
1. Department of Public Health, University of Helsinki, POB 41, FI 00014, FINLAND
2. University of Sydney, Sydney, Australia

Harri Hemilä
http://orcid.org/0000-0002-4710-307X
https://www.mv.helsinki.fi/home/hemila/
harri.hemila@helsinki.fi

version 2021-5-9

Supplement to an analysis published in:
Pharmacology Research & Perspectives
https://bpspubs.onlinelibrary.wiley.com/journal/20521707

Yellow colour is used to indicate the results that are shown in the main text

This supplement describes the extraction of common cold duration mortality data from figures 2 and 5 of the Koeninghofer IPD meta-analysis [28], and the statistical analysis.

> [28] Koenighofer M, Lion T, Bodenteich A, Prieschl-Grassauer E, Grassauer A, Unger H, Mueller CA, Fazekas T. Carrageenan nasal spray in virus confirmed common cold: individual patient data analysis of two randomized controlled trials. Multidiscip Respir Med. (2014) 9:57.
> https://doi.org/10.1186/2049-6958-9-57
> http://www.ncbi.nlm.nih.gov/pmc/articles/pmc4236476

Contents | **page**

Contents	page
Extraction of data on common cold duration for all virus-positive colds	2
Cox regression: all virus positive colds	5
Cox regression: two time periods of carrageenan effects	5
Analysis of coronavirus OC43 and 229E data	6
Analysis of influenza A data	9
Analysis of rhinovirus data	12
Quantile regression 95% CI for the 60th and 80th percentiles	15
Risk ratio for the common cold to last over 20 days and the NNT	16
Risk ratio for the lack of recurrence of cold symptoms	17
Calculation of the NNT for the lack of recurrence in the carrageenan patients	19

Extraction of data on common cold duration for all virus-positive colds

The recovery data in the IPD meta-analysis by Koenighofer [28] was published as survival curves. Their figure 2 for all virus-positive colds is copied below to illustrate the measurement of recovery over the follow-up period. Daily recovery is shown as steps in the survival curve. Both the ITT and per protocol analyses are shown in the original figure. Since the ITT analysis is generally preferable [30,31], we extracted the ITT data which is shown by gray in the figure.

When the number of patients is quite low, as in this case, it is possible to back-calculate from the survival curve the number of patients who recovered on each downward step. The size of the steps was measured from the digital figure as pixels and the scale of the figure as pixels was used to determine the number of recovered patients on each step. A spreadsheet was used to transform the pixel-values to the number of persons who recovered.

Similar approach was used to regenerate the data sets for the common cold episodes caused by coronaviruses, influenza A virus, and rhinoviruses which were published in figure 5 [28].

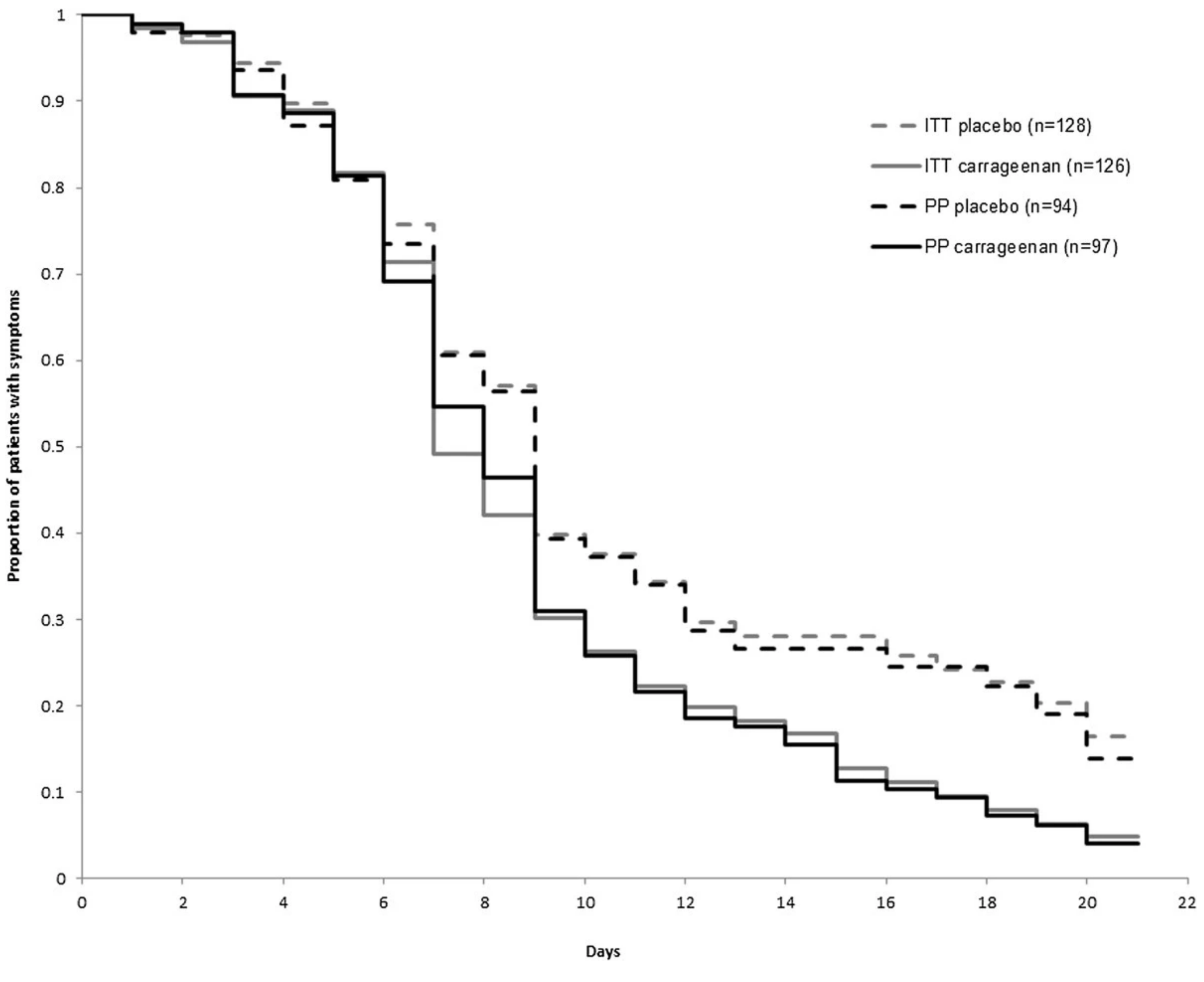

Table 1: Recovery from the common cold by carrageenan administration (from figure 2 of Koenighofer (2014) [28])

The left-hand side of this table shows the measurement of the steps in figure 2 [28].
The right-hand side shows the number of patients who recovered by the end of the given day.
The number of patients on each step can be inferred with great accuracy.

All virus-positive colds, Figure 2 [28]

**Scale**			**Carrag**	**Placebo**
Still sick	pixels	N:	126	128
100%	7			
0%	875			

gray:	line	dash	N cumulative Calculated		N of patients on each step Calculated		N of patients on each step Rounded to integers		
**Day**	**Carrag** pixels	**Placebo** pixels	**Carrag**	**Placebo**	**Carrag**	**Placebo**	**Carrag**	**Placebo**	**Day**
**0**	7	7	126.00	128.00	126.00	128.00	0	0	**0**
**1**	20	20	124.11	126.08	-1.89	-1.92	2	2	**1**
**2**	34	27	122.08	125.05	-2.03	-1.03	2	1	**2**
**3**	88	54	114.24	121.07	-7.84	-3.98	8	4	**3**
**4**	102	95	112.21	115.02	-2.03	-6.05	2	6	**4**
**5**	165	169	103.06	104.11	-9.15	-10.91	9	11	**5**
**6**	255	217	90.00	97.03	-13.06	-7.08	13	7	**6**
**7**	448	346	61.98	78.01	-28.02	-19.02	28	19	**7**
**8**	510	380	52.98	73.00	-9.00	-5.01	9	5	**8**
**9**	613	529	38.03	51.02	-14.95	-21.97	15	22	**9**
**10**	647	549	33.10	48.07	-4.94	-2.95	5	3	**10**
**11**	681	576	28.16	44.09	-4.94	-3.98	5	4	**11**
**12**	702	617	25.11	38.05	-3.05	-6.05	3	6	**12**
**13**	716	631	23.08	35.98	-2.03	-2.06	2	2	**13**
**14**	729	631	21.19	35.98	-1.89	0.00	2	0	**14**
**15**	764	631	16.11	35.98	-5.08	0.00	5	0	**15**
**16**	778	650	14.08	33.18	-2.03	-2.80	2	3	**16**
**17**	793	664	11.90	31.12	-2.18	-2.06	2	2	**17**
**18**	806	678	10.02	29.05	-1.89	-2.06	2	2	**18**
**19**	820	698	7.98	26.10	-2.03	-2.95	2	3	**19**
**20**	833	732	6.10	21.09	-1.89	-5.01	2	5	**20**
				**Cured by the end of day 20:**	119.90	106.91	120	107	
				**Total not cured by day 20:**	6.10	21.09	6	21	
						**No recovery:**	**6**	**21**	
						**Total N**	126	128	
						**Quantile no recovery:**	0.952	0.836	

Distribution of cold duration in all virus-positive patients by carrageenan administration

Total Observations in Table: 253

ALL$Day	ALL$Carrag 0	1	Row Total
1	2	2	4
2	1	2	3
3	4	8	12
4	6	2	8
5	11	9	20
6	7	13	20
7	19	28	47
8	5	9	14
9	22	15	37
10	3	5	8
11	4	5	9
12	6	3	9
13	2	2	4
14	0	2	2
15	0	5	5
16	3	2	5
17	2	2	4
18	2	2	4
19	3	2	5
20	25	8	33
Column Total	127	126	253

Cox regression: all virus positive colds

```
> ALL_cox   <- coxph(ALL_S ~ ALL$Carrag, method = "exact")
> summary(ALL_cox)
Call:
coxph(formula = ALL_S ~ ALL$Carrag, method = "exact")

  n= 253, number of events= 227
            coef exp(coef) se(coef)    z Pr(>|z|)
ALL$Carrag 0.429     1.536    0.146 2.94   0.0033 **

           exp(coef) exp(-coef) lower .95 upper .95
ALL$Carrag      1.54      0.651      1.15      2.05

Concordance= 0.543  (se = 0.02 )
Likelihood ratio test= 8.65  on 1 df,   p=0.003
Wald test            = 8.62  on 1 df,   p=0.003
Score (logrank) test = 8.7  on 1 df,   p=0.003
```

Cox regression: two time periods of carrageenan effects

```
> TwoPeriods_Cox <- coxph(Surv(tstart, tstop, event) ~
Carrag:strata(tgroup), data=TwoPeriods, method = "efron")
> TwoPeriods_Cox
Call:
coxph(formula = Surv(tstart, tstop, event) ~ Carrag:strata(tgroup),
    data = TwoPeriods, method = "efron")

                              coef exp(coef) se(coef)    z     p
Carrag:strata(tgroup)tgroup=1 -0.02      0.98     0.29 -0.1 0.938
Carrag:strata(tgroup)tgroup=2  0.50      1.64     0.15  3.3 0.001

Likelihood ratio test=11  on 2 df, p=0.004
n= 459, number of events= 227
> exp(confint(TwoPeriods_Cox))
                              2.5 % 97.5 %
Carrag:strata(tgroup)tgroup=1 0.552   1.73
Carrag:strata(tgroup)tgroup=2 1.221   2.21
```

Distribution of cold duration in coronavirus OC43 and 229E patients by carrageenan administration,

Data from Eva Prieschl-Grassauer (2020-11-10)

Day	Carrag	Placebo
**0**		
**1**	0	1
**2**	2	0
**3**	1	1
**4**	1	1
**5**	4	2
**6**	5	0
**7**	6	5
**8**	4	3
**9**	10	8
**10**	1	0
**11**	2	2
**12**	2	2
**13**	0	0
**14**	1	0
**15**	2	0
**16**	0	1
**17**	0	1
**18**	0	1
**19**	1	1
**20**	1	2
**Sums:**	43	31
**Total:**	45	43
**Not recovered:**	2	12

Total Observations in Table: 88

hCV$Day	hCV$Carrag 0	1	Row Total
1	1	0	1
2	0	2	2
3	1	1	2
4	1	1	2
5	2	4	6
6	0	5	5
7	5	6	11
8	3	4	7
9	8	10	18
10	0	1	1
11	2	2	4
12	2	2	4
14	0	1	1
15	0	2	2
16	1	0	1
17	1	0	1
18	1	0	1
19	1	1	2
20	14	3	17
Column Total	43	45	88

Cox regression: coronavirus OC43 and 229E colds

```
> hCV_coxE   <- coxph(hCV_S ~ hCV$Carrag, method = "exact")
> summary(hCV_coxE)
Call:
coxph(formula = hCV_S ~ hCV$Carrag, method = "exact")

  n= 88, number of events= 74

             coef exp(coef) se(coef)     z Pr(>|z|)
hCV$Carrag 0.8725    2.3929   0.2625 3.324 0.000886 ***
---


           exp(coef) exp(-coef) lower .95 upper .95
hCV$Carrag     2.393     0.4179     1.431     4.003

Concordance= 0.598  (se = 0.032 )
Likelihood ratio test= 11.3  on 1 df,   p=8e-04
Wald test            = 11.05  on 1 df,   p=9e-04
Score (logrank) test = 11.48  on 1 df,   p=7e-04

> lrtest(hCV_coxE)
Likelihood ratio test

Model 1: hCV_S ~ hCV$Carrag
Model 2: hCV_S ~ 1
  #Df  LogLik Df  Chisq Pr(>Chisq)
1   1 -192.92
2   0 -198.57 -1 11.297  0.0007762 ***

---
```

Distribution of cold duration in influenza A virus patients by carrageenan administration

InfA, Figure 5 [28]

**Scale**			**Carrag**	**Placebo**
Still sick	pixels	N:	23	24
100%	1051			
0%	4591			

	light gray line	dash	N cumulative Calculated		N of patients on each step Calculated		N of patients on each step Rounded to integers		
**Day**	**Carrag** pixels	**Placebo** pixels	**Carrag**	**Placebo**	**Carrag**	**Placebo**	**Carrag**	**Placebo**	**Day**
**0**	1051	1051	23.00	24.00	23.00	24.00	0	0	**0**
**1**	1199	1051	22.04	24.00	-0.96	0.00	1	0	**1**
**2**	1360	1051	20.99	24.00	-1.05	0.00	1	0	**2**
**3**	1666	1198	19.00	23.00	-1.99	-1.00	2	1	**3**
**4**	1818	1345	18.02	22.01	-0.99	-1.00	1	1	**4**
**5**	1968	1496	17.04	20.98	-0.97	-1.02	1	1	**5**
**6**	2283	1782	15.00	19.04	-2.05	-1.94	2	2	**6**
**7**	2743	2231	12.01	16.00	-2.99	-3.04	3	3	**7**
**8**	3043	2374	10.06	15.03	-1.95	-0.97	2	1	**8**
**9**	3353	2967	8.04	11.01	-2.01	-4.02	2	4	**9**
**10**	3513	3109	7.00	10.05	-1.04	-0.96	1	1	**10**
**11**	3666	3109	6.01	10.05	-0.99	0.00	1	0	**11**
**12**	3666	3263	6.01	9.00	0.00	-1.04	0	1	**12**
**13**	3666	3263	6.01	9.00	0.00	0.00	0	0	**13**
**14**	3968	3263	4.05	9.00	-1.96	0.00	2	0	**14**
**15**	4275	3263	2.05	9.00	-1.99	0.00	2	0	**15**
**16**	4436	3405	1.01	8.04	-1.05	-0.96	1	1	**16**
**17**	4436	3549	1.01	7.06	0.00	-0.98	0	1	**17**
**18**	4436	3549	1.01	7.06	0.00	0.00	0	0	**18**
**19**	4436	3549	1.01	7.06	0.00	0.00	0	0	**19**
**20**	4591	3854	0.00	5.00	-1.01	-2.07	1	2	**20**
			**Cured by the end of day 20:**		23.00	19.00	23	19	
			**Total not cured:**		0.00	5.00			

Total Observations in Table: 47

InfA$Day	InfA$Carrag 0	1	Row Total
1	0	1	1
2	0	1	1
3	1	2	3
4	1	1	2
5	1	1	2
6	2	2	4
7	3	3	6
8	1	2	3
9	4	2	6
10	1	1	2
11	0	1	1
12	1	0	1
14	0	2	2
15	0	2	2
16	1	1	2
17	1	0	1
20	7	1	8
Column Total	24	23	47

Cox regression: influenza A colds

```
> InfA_cox   <- coxph(InfA_S ~ InfA$Carrag, method = "exact")
> summary(InfA_cox)
Call:
coxph(formula = InfA_S ~ InfA$Carrag, method = "exact")

  n= 47, number of events= 42

             coef exp(coef) se(coef)    z Pr(>|z|)
InfA$Carrag 0.785     2.192    0.343 2.29    0.022 *
---


            exp(coef) exp(-coef) lower .95 upper .95
InfA$Carrag      2.19      0.456      1.12      4.29

Concordance= 0.582  (se = 0.043 )
Likelihood ratio test= 5.34  on 1 df,   p=0.02
Wald test            = 5.24  on 1 df,   p=0.02
Score (logrank) test = 5.44  on 1 df,   p=0.02

> lrtest(InfA_cox)
Likelihood ratio test

Model 1: InfA_S ~ InfA$Carrag
Model 2: InfA_S ~ 1
  #Df LogLik Df Chisq Pr(>Chisq)
1   1   -104
2   0   -106 -1  5.34      0.021 *
```

Distribution of cold duration in rhinovirus patients by carrageenan administration,

Data from Eva Prieschl-Grassauer (2020-11-1)

Day	Carrag	Placebo
**1**	1	2
**2**	0	1
**3**	7	2
**4**	2	4
**5**	5	7
**6**	6	6
**7**	18	11
**8**	3	2
**9**	7	13
**10**	4	3
**11**	2	2
**12**	0	4
**13**	2	2
**14**	1	0
**15**	3	0
**16**	1	1
**17**	2	2
**18**	2	1
**19**	1	2
**20**	0	1
**Sums:**	67	66
**Total:**	**70**	**80**
**Not recovered:**	3	14

Total Observations in Table: 150

hRV$Day	hRV$Carrag 0	1	Row Total
1	2	1	3
2	1	0	1
3	2	7	9
4	4	2	6
5	7	5	12
6	6	6	12
7	11	18	29
8	2	3	5
9	13	7	20
10	3	4	7
11	2	2	4
12	4	0	4
13	2	2	4
14	0	1	1
15	0	3	3
16	1	1	2
17	2	2	4
18	1	2	3
19	2	1	3
20	15	3	18
Column Total	80	70	150

Cox regression: rhinovirus colds

```
> hRV_cox   <- coxph(hRV_S ~ hRV$Carrag, method = "exact")
> summary(hRV_cox)
Call:
coxph(formula = hRV_S ~ hRV$Carrag, method = "exact")

  n= 150, number of events= 128

             coef exp(coef) se(coef)    z Pr(>|z|)
hRV$Carrag 0.533     1.705    0.192 2.77   0.0056 **
---
Signif. codes:  0 ‘***’ 0.001 ‘**’ 0.01 ‘*’ 0.05 ‘.’ 0.1 ‘ ’ 1

           exp(coef) exp(-coef) lower .95 upper .95
hRV$Carrag       1.7      0.587      1.17      2.49

Concordance= 0.553  (se = 0.026 )
Likelihood ratio test= 7.68  on 1 df,   p=0.006
Wald test            = 7.68  on 1 df,   p=0.006
Score (logrank) test = 7.8  on 1 df,   p=0.005

> lrtest(hRV_cox)
Likelihood ratio test

Model 1: hRV_S ~ hRV$Carrag
Model 2: hRV_S ~ 1
  #Df LogLik Df Chisq Pr(>Chisq)
1   1   -356
2   0   -360 -1  7.68     0.0056 **
```

Quantile regression 95% CI for the 60th and 80th percentiles, the latter to Figure 2

```
> fit.crq=crq(ALL_S ~ ALL$Carrag, tau= 0.5, method="PengHuang")
> summary.crq(fit.crq, c(0.6,0.8), alpha = .05, R = 1000)

tau: [1]  0.6

Coefficients:
             Value   Lower Bd Upper Bd Std Error T Value Pr(>|t|)
(Intercept) 10.000   6.552   14.172    1.944      5.145   0.000
ALL$Carrag  -1.000  -8.619    2.448    2.823     -0.354   0.723

tau: [1] 0.8
   Number of NA Bootstrap Replications:  6 out of 1000
Coefficients:
             Value    Lower Bd Upper Bd Std Error T Value Pr(>|t|)
(Intercept)  20.000  16.552    20.000    0.880     22.740   0.000
ALL$Carrag   -6.395 -10.567    -2.948    1.944     -3.290   0.001
```

Risk ratio for the common cold to last over 20 days and the NNT

For the extraction of 6 and 21, see pages 2 and 3 of this supplement

```
> riskratio(6,21,126,128)
           Disease Nondisease Total
Exposed          6        120   126
Nonexposed      21        107   128

        Risk ratio estimate and its significance probability

data:  6 21 126 128
p-value = 0.003
95 percent confidence interval:
 0.121 0.695
sample estimates:
[1] 0.29
```

Calculation of the NNT

```
>
> prop.test(c(6, 21),c(126, 128),correct=FALSE)

        2-sample test for equality of proportions without continuity correction

data:  c(6, 21) out of c(126, 128)
X-squared = 9, df = 1, p-value = 0.003
alternative hypothesis: two.sided
95 percent confidence interval:
 -0.1906 -0.0423
sample estimates:
prop 1 prop 2
0.0476 0.1641

>
> (Difference = 0.16406250 - 0.04761905)
[1] 0.116
> (NNT = 1/Difference)
[1] 8.59
>
> # 95% CI for NNT
>
> (1/0.04229085)
[1] 23.6
> (1/0.19059605)
[1] 5.25
```

Risk ratio for the lack of recurrence of cold symptoms

Extraction of data

figure 3 [28]

**Relapses**	**Carrageenan**	**Placebo**	**RR**
ALL IPD			
N	126	128	
Reported %	13%	29%	
Relapses	16.38	37.12	
Rounded	16	37	0.44

figure 6 [28]

**Relapses**	**Carrageenan**	**Placebo**	**RR**
**hCV**			
N	45	43	
Reported %	18%	44%	
Relapses	8.1	18.92	
Rounded	8	19	0.40
**InfA**			
N	23	24	
Reported %	13%	38%	
Relapses	2.99	9.12	
Rounded	3	9	0.35
**hRV**			
N	70	80	
Reported %	9%	20%	
Relapses	6.3	16	
Rounded	6	16	0.43

```
> # ALL
> riskratio(16, 37, 126, 128)
           Disease Nondisease Total
Exposed         16        110   126
Nonexposed      37         91   128

        Risk ratio estimate and its significance probability

data:  16 37 126 128
p-value = 0.002
95 percent confidence interval:
 0.258 0.748
sample estimates:
[1] 0.439

> # hCV
> riskratio(8, 19, 45, 43)
           Disease Nondisease Total
Exposed          8         37    45
Nonexposed      19         24    43

        Risk ratio estimate and its significance probability

data:  8 19 45 43
p-value = 0.008
95 percent confidence interval:
 0.197 0.820
sample estimates:
[1] 0.402

> # InfA
> riskratio(3, 9, 23, 24)
           Disease Nondisease Total
Exposed          3         20    23
Nonexposed       9         15    24

        Risk ratio estimate and its significance probability

data:  3 9 23 24
p-value = 0.057
95 percent confidence interval:
 0.10743 1.12612
sample estimates:
[1] 0.34783

> # hRV
> riskratio(6, 16, 70, 80)
           Disease Nondisease Total
Exposed          6         64    70
Nonexposed      16         64    80

        Risk ratio estimate and its significance probability

data:  6 16 70 80
p-value = 0.049
95 percent confidence interval:
 0.17746 1.03503
sample estimates:
[1] 0.42857
```

Calculation of the NNT for the lack of recurrence in the carrageenan patients

```
> prop.test(c(16, 37),c(126, 128),correct=FALSE)

        2-sample test for equality of proportions without continuity correction

data:  c(16, 37) out of c(126, 128)
X-squared = 10, df = 1, p-value = 0.001
alternative hypothesis: two.sided
95 percent confidence interval:
 -0.2598 -0.0644
sample estimates:
prop 1 prop 2
 0.127  0.289

>
> (Difference = 0.289 - 0.127)
[1] 0.162
> (NNT = 1/Difference)
[1] 6.17
>
> # 95% CI for NNT
>
> (1/0.2598)
[1] 3.85
> (1/0.0644)
[1] 15.5
```
